# Probing 3D Molecular Orientation and Crystallization Behavior in PLLA/PEG Spherulites Using O‑PTIR

**DOI:** 10.1021/acs.analchem.5c07255

**Published:** 2026-03-25

**Authors:** Karolina Kosowska, Honorata Oles, Tomasz P. Wrobel

**Affiliations:** † Solaris National Synchrotron Radiation Centre, 37799 Jagiellonian University, Czerwone Maki 98, PL30392 Kraków, Poland; ‡ Doctoral School of Exact and Natural Sciences, Jagiellonian University, Prof. St. Łojasiewicza St 11, PL30348 Kraków, Poland

## Abstract

Infrared imaging (IR), in addition to chemical specificity, has recently gained the capability to retrieve 3D molecular orientation information. When combined with IR super-resolution optical photothermal infrared (O-PTIR) spectroscopy, it enables unprecedented tracking of the three-dimensional macromolecular organization of complex layer systems in PLLA spherulites. The use of linearly polarized IR excitation, combined with a rapid computational algorithm applied to two nonparallel absorption bands, allowed tracing different paths of spherulite formation in PLLA/PEG films depending on the crystallization conditions. The plasticizer facilitated polymer crystallization but also caused disruption of molecular organization and a second crystallization, leading to lamellae branching. High-resolution O-PTIR microscopy, independent of the IR diffraction limit, does not require time-consuming sample preparation and, in combination with 3D orientation determination, is an excellent tool in studies on structural changes and morphology-property relationships in biology and materials science.

## Graphical abstract

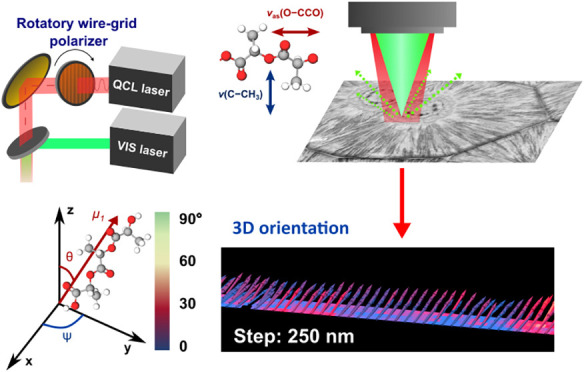

## Introduction

In the past few years, optical photothermal infrared (O-PTIR) microscopy has become a widely used method, mainly in biological^1–3^ and polymer^4,5^ studies. It surpasses classical fourier-transform infrared (FT-IR) microscopy in terms of spatial resolution, not dependent on the IR diffraction limit, and with a small contribution of water signal. The visible beam probe detects the change in photothermal chemical contrast induced by IR absorption. When a wavelength quantum cascade laser (QCL) induces peak absorption in the sample, a local temperature increase occurs, resulting in sample thermal expansion and a change in refractive index.^6^ Photothermal contrast can be obtained by measuring the change in divergence in the copropagating probe beam; however, other geometries are also available. Since the spectral resolution depends on the wavelength of the visible probe, it is constant over the entire spectral range. Water artifacts are absent or minimal, due to the small temperature increase and the high heat capacity. In the copropagation mode, substrates intended for IR measurements are not necessary.

Advanced infrared microscopy methods can provide much broader information than just chemical composition and morphology imaging. By using polarized O-PTIR microscopy, we obtain information about the orientation of molecules in three-dimensional space and the degree of their self-organization with submicron resolution, entering the field previously dominated by X-ray methods, which for organic samples use synchrotron radiation.^7,8^ The developed method of 3D angles calculation is comprehensive and easy to reproduce for various types of samples, including biological materials such as tissues,^9,10^ and polymers.^11–13^ Precise determination of molecular orientation with high spatial resolution is critical for understanding the processes occurring in organic materials such as the relationships between their structure and properties. Due to the dependence of IR absorption on polarization, it is possible to determine the orientation of the molecule in 3D space. Two anisotropic vibrational modes, perpendicular to each other, are independently analyzed.^14^

The crystallization and aggregation process leads to the formation of organized structures. Characteristic of polymers, spherulites are normally built of indistinct lamellae growing radially from the nucleation center. Many factors can influence the morphology of polymer and lamella packing in the spherulite. Taking poly­(l-lactic acid) (PLLA) as an example, several works revealed dependence between spherulite forms and the temperature of crystallization,^15^ PLLA/second polymer ratio,^16,17^ molecular weight,^18^ and others. With the use of polarized optical microscopy (POM) and atomic force microscopy (AFM), various lamella packing patterns in PLLA have been identified. In their classic form, lamellae have the shape of plates that create stacks that often look like flat terrace structures. Periodic structures in the form of rings (so-called banded spherulites) have been found in many polymers,^19–21^ although their origin is still a matter of debate. In the case of PLLA, the crystalline patterns are even richer. Branched dendritic structures have been observed in blends with other polymers.^22,23^ Although microscopic observations provide very high spatial resolution, the information about the molecular orientation is not direct. In our previous proof-of-concept work, we demonstrated that spatial high-resolution linearly polarized O-PTIR microscopy can be effectively used to determine the three-dimensional orientation of molecules in a polymer spherulite.^12^ The molecular alignment results obtained were consistent with FT-IR microscopy, for which the four-polarization method was originally developed. In the present work, we introduced an external plasticizer in the form of low-molecular-weight poly­(ethylene oxide) (PEG) to the PLLA and used different temperatures of crystallization. Using a new tool, we investigated the three-dimensional organization of macromolecules in three morphologically distinct structures. This new approach allows for the correlation of morphology with the three-dimensional packing of macromolecules. The studies demonstrated that the proposed method is not only highly resolving but also sensitive to subtle changes in crystal assembly.

The use of a modified experimental O-PTIR system and a 3D orientation algorithm allowed for the visualization of structure and the discovery of details of crystallization processes, such as side branching and second crystallization with submicron resolution.

## Experimental Section

### Sample Preparation

Poly­(l-lactic acid) (PLLA) with a low content (1.4%) of d-lactide was purchased from IngeoTM Biopolymer. Chloroform was used for analysis (99.8%), amylene stabilized was obtained from Idalia Sp. J (Radom, Poland). The poly­(ethylene glycol) (PEG) with an average molar mass of 4000 g/mol, used as a plasticizer, was obtained from Merck Life Science Sp. z o.o. (Poznań, Poland). A suitable composition polymer/plasticizer of PLLA/PEG (70/30) at a concentration of 0.5% by weight was prepared via the solution blending method.^24^ The drops of solution were cast onto three calcium fluoride (CaF_2_) substrates and left in the fume hood for evaporation for 24 h at room temperature.^25,26^

The PLLA-based polymers typically have two temperatures of crystallization, the first, after glass transition, and the second near the melting temperature.^25,26^ To study the influence of annealing temperature on molecule organization in crystalline forms, crystallization was carried out under three different conditions. First, three PLLA/PEG films were slowly heated on hot stages to the melting temperature (163 °C) and kept for 15 min to eliminate the pre-existing crystal structures. Next, after slowly cooling to the desired temperature, the films were annealed under the following conditions:

1. Annealing for 15 min at the second temperature (132 °C) for isothermal crystallization (Spherulite Type 1)
2. Annealing in nonisothermal conditions (1 h) – annealing for 5 min at 132 °C, slow cooling, annealing at 86 ^o^C for 15 min (Spherulite Type 2)
3. Annealing for 15 min at a low temperature (86 °C) for isothermal crystallization (Spherulite Type 3)

Additionally, the model PLLA sample without PEG addition was prepared following the same steps. The film, after melting (165 °C), was annealed for 15 min at the second temperature of crystallization (150 °C).

For the PLLA/PEG blend, the addition of a low-molecular-weight polymer decreased the phase transition temperatures. The melting temperature and the first and second crystallization temperatures for PLLA and PLLA/PEG were determined based on DSC curves (Figure 1a).

**Figure 1:**
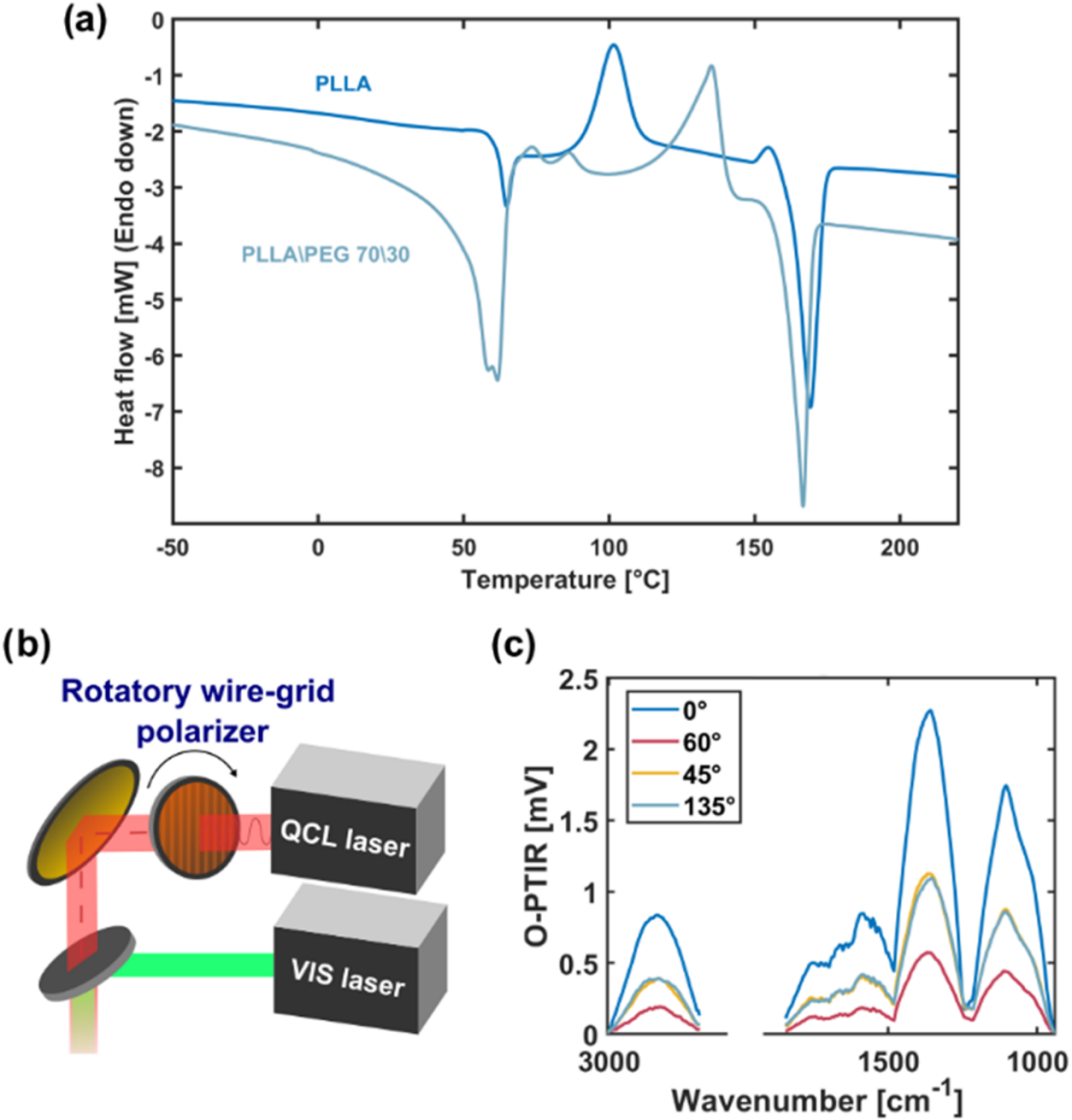
Differential scanning calorimetry (DSC) curves of PLLA and PLLA/PEG (a), scheme of the O-PTIR laser beam path with a rotary wire-grid polarizer (b), and plots of background for four angles of polarizations (c).

### O-PTIR Polarization-Controlled Imaging

Imaging was done using a mIRage O-PTIR microscope. A quantum cascade laser (QCL) was used as a pump beam with a pulse rate of 100 kHz in a 1% duty cycle, and a green laser (532 nm) as a probe beam. Images were collected at two frequencies: 1094 and 1044 cm^–1^. Measurements were performed in the co-propagation mode using a standard detector and a 40×, 0.78 NA Schwarzschild IR objective with an 8 mm working distance. Intensity maps for four different polarization angles of IR were collected with a step of 250 nm and a scan rate of 1.6 Hz. The background was measured separately for every angle of polarization, on a low-e slide (Kevley Technologies). A wire-grid polarizer was used to control the natural polarization direction of the QCL laser (Figure 1b). Four polarizations of interest (−45 (135), 0, 45, and 60°) were achieved by the rotation of the polarizer. The powers of the QCL and probe beam laser were adjusted for every sample separately. Since the polarizer rotation reduces the QCL intensity, the signal was background-corrected (Figure 1c).

## Results and Discussion

### Four-Polarization O-PTIR

DC images (collected probe beam DC intensity at the detector) of four films measured with 1094 cm^–1^ are shown in Figure 2a–d. Poly­(l-lactic acid) typically exhibits two temperatures of crystallization: first crystallization after glass transition, and second crystallization before melting. The DSC heat scans of PLLA and PLLA/PEG can be found in Figure 1a. A plasticizer can accelerate the growth of spherulites by increasing chain mobility, lowering the glass transition temperature.^27^ Crystallization of pure PLLA at high temperature (150 °C) provokes the formation of well-structured small spherulites. Naturally low nucleation density resulted in a low number of spherulites surrounded by the amorphous phase (Figure 2a). The addition of PEG results in the formation of spherulites that are three to five times larger and have irregular rings (Figure 2b, Type 1). It suggests the plasticizer was well incorporated into the PLLA matrix. Crystallization at low temperature strongly impacted the morphology. The next sample, PLLA/PEG (Type 2), was annealed at a high temperature (132 °C) for 5 min, then slowly cooled to the second temperature (86 °C) and annealed for 15 min. Nonisothermal conditions increased the dynamics of spherulite growth, which filled the free space. The two phases of growth are reflected in the morphology, characterized by dense, atypical rings surrounding the center of spherulite growth and radially growing lamellae (Figure 2c). The first crystallization occurs just above the glass transition, promoting many nucleation sites from the amorphous phase, in contrast to high temperature near the melting point (Type 3). Figure 2d shows the PLLA/PEG film after isothermal crystallization at 86 °C. Similar to the previous sample, spherulites filled the space; however, they became smaller due to the greater number of nucleation centers and collisions.

**Figure 2:**
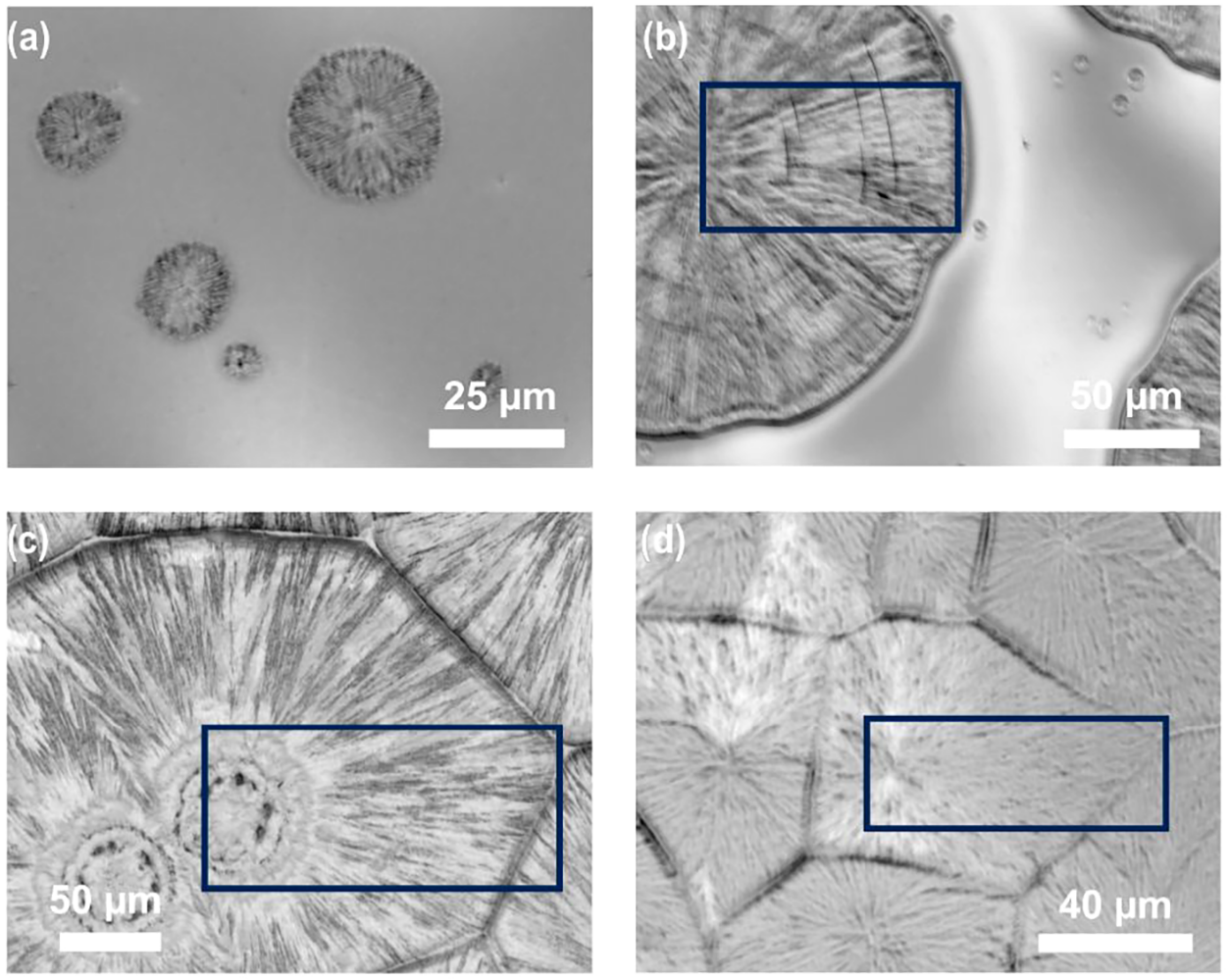
DC images for 1094 cm^–1^: PLLA crystallized at 150 °C (a), PLLA/PEG annealed at second crystallization temperature (b), PLLA/PEG annealed at nonisothermal condition (c), PLLA/PEG annealed at first crystallization temperature (d).

To analyze the impact of crystallization conditions on lamellae assembly and the spherulite mechanism of growth, the polarization-controlled O-PTIR was performed. The typical spectrum of PLLA collected in co-propagation mode is presented in Figure 3a. The asymmetric stretching *ν*_as_(O–CCO) + ω­(CH_3_) mode at 1094 cm^–1^ is assigned as parallel^28,29^ to the main chain of the molecule and represents its direction in two-dimensional space. The azimuthal angle (ψ) can be easily calculated by employing the dependence of infrared absorbance on the direction of linearly polarized light. The absorptance reaches maximal values when the directions of the transition dipole moment and the linear polarization are parallel. The organization in the material can be described by the Hermans orientation function (*f*). The first assumption is that the polarizability of a chain segment can be described by a component parallel to the axis and a component perpendicular to it.^30^ The chain orientation with respect to the coordinate system is shown in Figure 3b. The Hermans function can be considered as a function of the probability of finding a molecule in a specific orientation. This orientation distribution function (ODF) can be expressed as a sum of the generalized Legendre functions. The second-order, associated with the polarized IR technique, quantifies the chain orientation:

1 $$\left\langle P_{2}\right\rangle =\frac{1}{2}\left(3\left\langle \cos^{2}\beta -1\right\rangle \right)$$

This factor is equal to 0 when the distribution is isotropic, and ranges from 1 when all the chains are aligned parallel to the reference direction, to −1/3 when all the chains are perpendicular to it. Two independent transition dipole moments, the first representing the main chain axis of the polymer, and the second orthogonal to μ_1_, can be used to calculate the 3D molecular orientation. As already mentioned, IR absorbance is polarization dependent.^31^ If we measure a given set for several angles of linear polarization, it is possible to fit the absorption-polarization dependence function for each vector separately and determine the maxima. We can express the rotation of the molecule in Euclidean space, using three angles: the azimuthal (ψ), axial (θ), and rotational (ϕ). The absorptance of the primary and secondary transition dipole moments can be expressed by Legendre polynomial coefficients used for the ODF.^14^

First, absorptance α_1_ for the first vector μ_1_ can be expressed as

2 α1(η)α1o=12[1−sin2⁡θ⁡cos2(η−ψ)]+13[3⁡sin2⁡θ⁡cos2(η−ψ)−1]x∫0πcos2⁡β⁡ρ(β)sin⁡βdβ

With the use of (1), the absorptance can be expressed as Legendre polynomial coefficients

3 $$\frac{\alpha_{1}\left(\eta \right)}{\alpha_{1}^{o}}=\left\langle P_{2}\right\rangle \sin^{2}\theta \cos^{2}\left(\eta -\psi \right)+\frac{1}{3}\left(1-\left\langle P_{2}\right\rangle \right)$$

Similarly, for the second μ_2_ vector

4 α2(η)α2o=−12{[cos⁡θ⁡cos⁡ϕ⁡cos(η−ψ)+sin⁡ϕ⁡sin(η−ψ)]2−1}+12{3[cos⁡θ⁡cos⁡ϕ⁡cos(η−ψ)+sin⁡ϕ⁡sin(η−ψ)]2−1}x∫0πcos2⁡β⁡ρ(β)sin⁡βdβ

5 $$\frac{\alpha_{2}\left(\eta \right)}{\alpha_{2}^{o}}=\left\langle P_{2}\right\rangle {\left[\cos\theta \cos\phi \cos\left(\eta -\psi \right)+\sin\phi \sin\left(\eta -\psi \right)\right]}^{2}+\frac{1}{3}\left(1-\left\langle P_{2}\right\rangle \right)$$

where α is the absorptance, η is the angle of polarization, and α°contains nonorientational quantities. The result is two sets of out-of-plane orientation angles (θ, φ) that are symmetrically mirrored concerning the *xy* plane. The first solution pair is set as 0 ≤ θ_1_≤ π/2 and 0 ≤ φ_1_≤ π, the second pair is its mirror image, as θ_2_ = π – θ_1_ and φ_2_ = −φ_1_. The theoretical details of the concurrent analysis of infrared polarization can be found in Lee’s publications.^14,32^

Applying the four-polarization method provides a map of the molecule’s orientation with additional information about the level of organization ⟨*P*_2_⟩. The single PLLA spherulite from Figure 2a was chosen for the analysis as the example (Model Spherulite). Figure 3c shows the DC images with part of the 3D orientation map overlaid on top. The color of a rod indicates the value of ⟨*P*_2_⟩. All maps were collected in co-propagation mode with a step of 250 nm. High spatial resolution allowed the capture of some of the morphological details. The orientation analysis exposes the nature of both phases in the polymer. The completely smooth and homogeneous amorphous phase in the DC image is composed of randomly organized molecules without oriented microdomains. In the spherulite, chains are mostly oriented almost perpendicular to the axis of the growth of the lamella, which is typical. The empty spaces are filled with amorphous phases. The order parameters ⟨*P*_2_⟩ reached their maximum values around 0.4; however, for well-formed spherulites, values between 0.7 and 0.8 were reported.^33^ We can assume that crystallization from melting resulted in low nucleation density. The low supercooling also contributes to the fact that small clusters do not reach critical size to be activated into nuclei^34^ and that the crystallization driving force is weak. Figure 3d shows histograms of ⟨*P*_2_⟩ of PLLA film for the amorphous phase and spherulites separately. The results are similar to those obtained by FT-IR in our previous publication.^11^ The order ⟨*P*_2_⟩ had values of 0.05 ± 0.01 and 0.11 ± 0.01 for the amorphous and crystalline phases, respectively. Even though the O-PTIR uses different phenomena to obtain the IR signal, the photothermal expansion, the methodology can be successfully transferred. Figure 3e shows the in-plane angle (ψ_r_) relative to the radial axes of the green line marked in Figure 3c. For the spherulite area, the values remain close to 100^o^, indicating that the projection of macromolecules in Figure 3c is almost perpendicular to the direction of lamellae growth.

**Figure 3:**
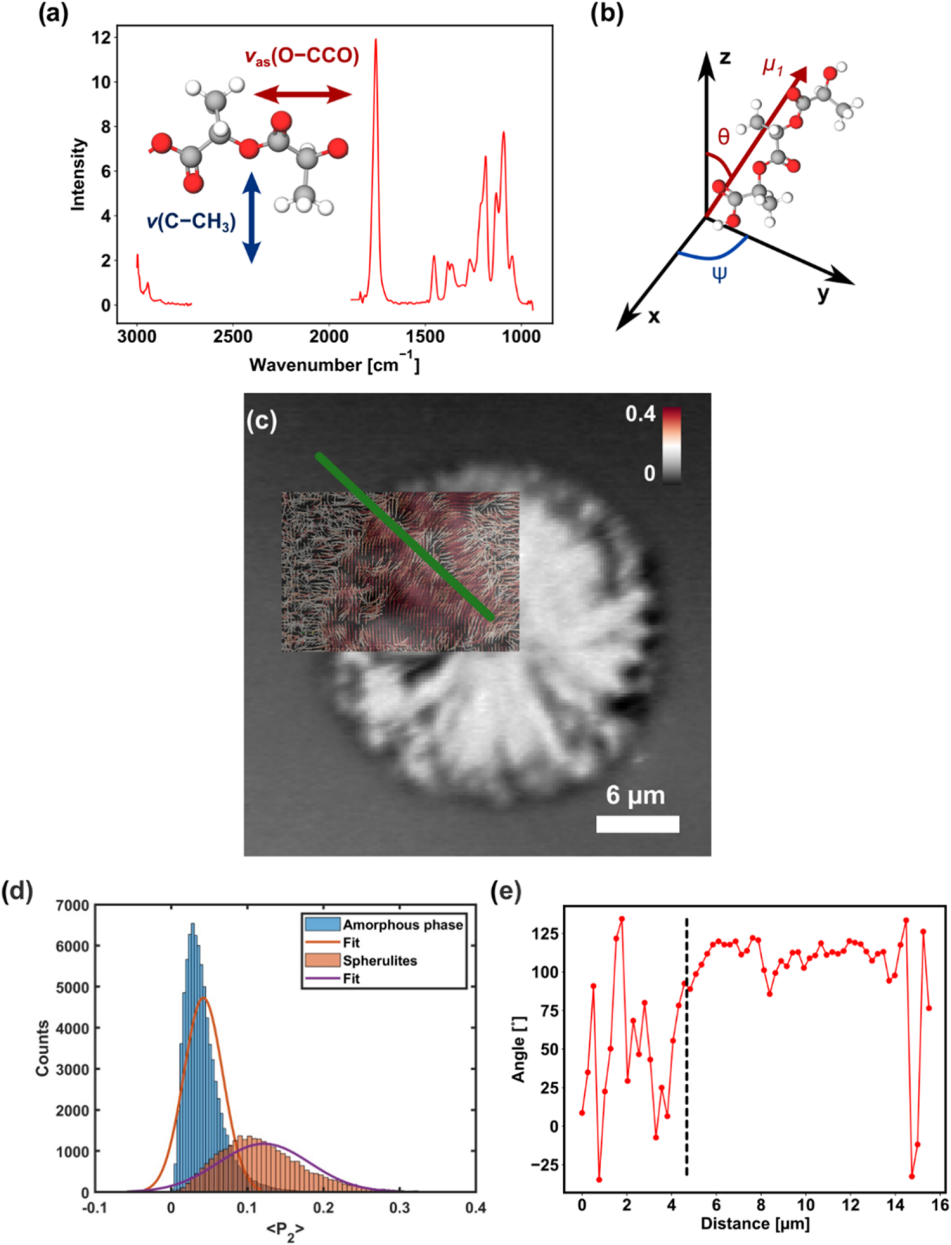
Representative O-PTIR spectrum of PLLA and scheme of the molecule with primary (*v*_as_(C–O–C) with ρ­(CH_3_)) and secondary (*v*(C–CH_3_)) vectors (a). Vectors defining molecular orientation in a Cartesian coordinate system: azimuthal (ψ), axial (θ), and rotational (ϕ, not included in the scheme) angles. (b). DC map of PLLA spherulite with superimposed illustration of 3D chains. The color of a rod indicates ⟨*P*_2_⟩ values (c). Histogram of the ⟨*P*_2_⟩ in the amorphous phase and spherulites (d). Relative ψ_r_ angle plot for the green line marked on the spherulite DC image (e).

### Three-Dimensional Images of Chain Orientation

Figure 2a–d illustrates three different spherulite patterns resulting from the crystallization of PLLA in the presence of low-molecular-weight PEG: round spherulite (Type 1), half-ring/half-lamellar branched (Type 2), and lamellar branched (Type 3). In the next section, these structures will be discussed in turn. The low-molecular-weight poly­(ethylene glycol) was applied as a plasticizer to enhance the crystallization process. Polyesters with a molecular structure similar to PLLA can be integrated into the polymer matrix as an external plasticizer. The most popular theory about the plasticizer suggests that the low-weight molecules weaken their secondary bonding effects by filling the space between polymer macromolecules.^35^ The diffusion of small plasticizer molecules and integration between long polymer chains leads to a weakening of the interactions between them and increased mobility.

The introduction of PEG lowered the temperatures of both crystallizations, indicating a plasticizer effect. The first sample was annealed at a high temperature of crystallization, which dropped from 150 to 134 °C. Compared to pure PLLA, the introduction of a plasticizer induced the growth of isolated large, circular spherulites (Type 1), with a diameter exceeding 100 μm (Figure 2b). It suggests that modification did not provide a significant change in nucleation density, but facilitated the diffusion of chains from the amorphous phase to the growing spherulite. To study the effect of PEG on the crystallization behavior of PLLA, the 4P–3D orientation was applied. For all calculations, as the primary, parallel vector, the asymmetric stretching *ν*_as_(O–CCO) + ω­(CH_3_) mode at 1094 cm^–1^ was used, and the *ν*(C–CH_3_) mode at 1044 cm^–1^ as the perpendicular.^12^

Figure 4a shows the ψ map for the region marked with a dark blue rectangle in the DC image, with the area from the center of nucleation to the edge of the spherulite. At first glance, the arrangement seems typical. The macromolecular alignment along the lamella growth was close to 90°. Characteristic intermittent rings were visible both in the DC images and in the orientation map. Spherulites with rings are a common phenomenon for poly­(lactic acid). Periodic morphology usually results from the self-organization of the lamella assembly into repetitive, twisted structures.^33,36,37^ To investigate the nature of the rings, the 3D orientation of the chains between the two rings was illustrated using rods along the pink section (Figure 4a,b). The rod’s color indicates the out-of-plane angle. The 3D illustration reveals that in-plane, the chains are aligned perpendicularly to the spherulite growth axis along the entire length, while out-of-plane, the angle oscillates very slightly. The chains lie almost flat on the substrate, but the maximum is reached in the middle, between rings. The mechanism of lamella folding and crack formation here is different than lamella twist.

**Figure 4:**
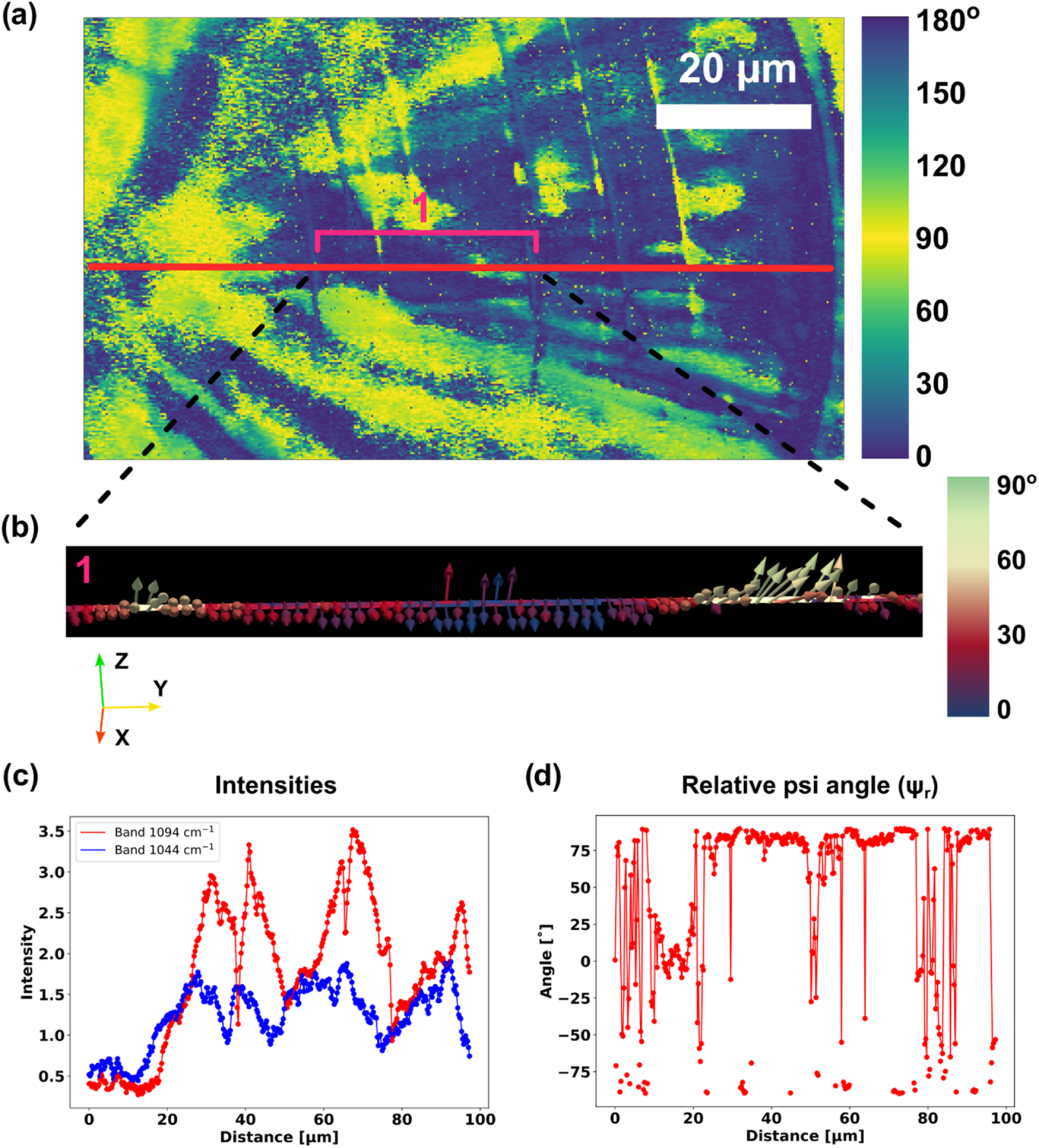
Illustrations of chain orientations of PLLA/PEG annealed at the second crystallization temperature. Azimuthal angle (ψ) map of the region marked with a rectangle in Figure 2b (a). 3D orientation of chains along the red line, limited to region 1. The color of the rod indicates tilt (b). Intensity plots of 1094 and 1044 cm^–1^ bands for polarization 0° (horizontal, c) and ψ angle for lines marked on psi maps (d).

Figure 4c shows the IR intensity profiles of both modes along the red line. They exhibited periodicity after passing the amorphous nucleation center. The spectra were prepared for 90° polarization, the vertical one. There was agreement on the modes; four peaks were separated by cracks, with intensity maxima between them. This suggests some periodicity and, at the same time, irregularity in spherulite growth. It should be emphasized that this is a dynamic process, during which the temperature and access to the primary amorphous phase necessary for the continuation of spherulite formation change locally. The crack-like character of the rings is also suggested by the jumping of ψ values, indicating a scattering effect (Figure 4d). Except for the nucleation center, the Ψ_r_ values oscillated around 90°. Near the rings, the discontinuities appeared, not the smooth transition that should be expected for lamella twist. The scattering effect is the source of signal and polarization anisotropy reduction, which, in consequence, provides false values of outputs (*ψ, θ,* ⟨P2⟩) for the orientation-angles algorithm.

Based on the results, we can draw some general hypotheses about the kinetics of spherulite growth and lamella organization, but the real understanding of the process requires extending the methodology to include *in situ* studies. We can consider several causes of cracks that are not the result of lamella twisting. The first may be the cyclic growth of the spherulite due to the depletion of the local amorphous phase and subsequent diffusion from further areas. Another factor worth considering is the depletion of the plasticizer. PEG was introduced as an external plasticizer by the blending method, and unlike internal plasticizers, it was not bonded to the polymer matrix. The migration of the plasticizer can reduce the elasticity, leading to material cracks. At this point, it should be noted that the mechanical properties of the film and interactions with the substrate resulted in large fluctuations in thickness.

Second PLLA/PEG sample (Type 2), after annealing at 134 °C, was subjected to additional crystallization by cooling to the first crystallization temperature at 86 °C. Figure 2c shows a DC image of the film with neighboring, large spherulites filling the entire free space. Wide rings resembling nests were visible around the center of the spherulites. The in-plane orientation map (Figure 5a) revealed the details of the chain in-plane orientation. What is more, in the nest, the molecules showed a different type of organization. In combination with the relative ψ_r_ angle plot from Figure 5d, we can conclude that the organization is quite discrete. The graph shows the angle of the chains to the red line. For the nest region, the values oscillate around 20°, which means chain alignment was closer to the parallel orientation to the line and the theoretical axis of lamella growth, than to the typical perpendicular one. The first part of Figure 5b shows the 3D orientation of macromolecules with rods used for the nest fragment marked with an orange segment. The angle between the in-plane *XY* surface and the rod exceeded 45° in many places, and the chain did not align flat on it.

**Figure 5:**
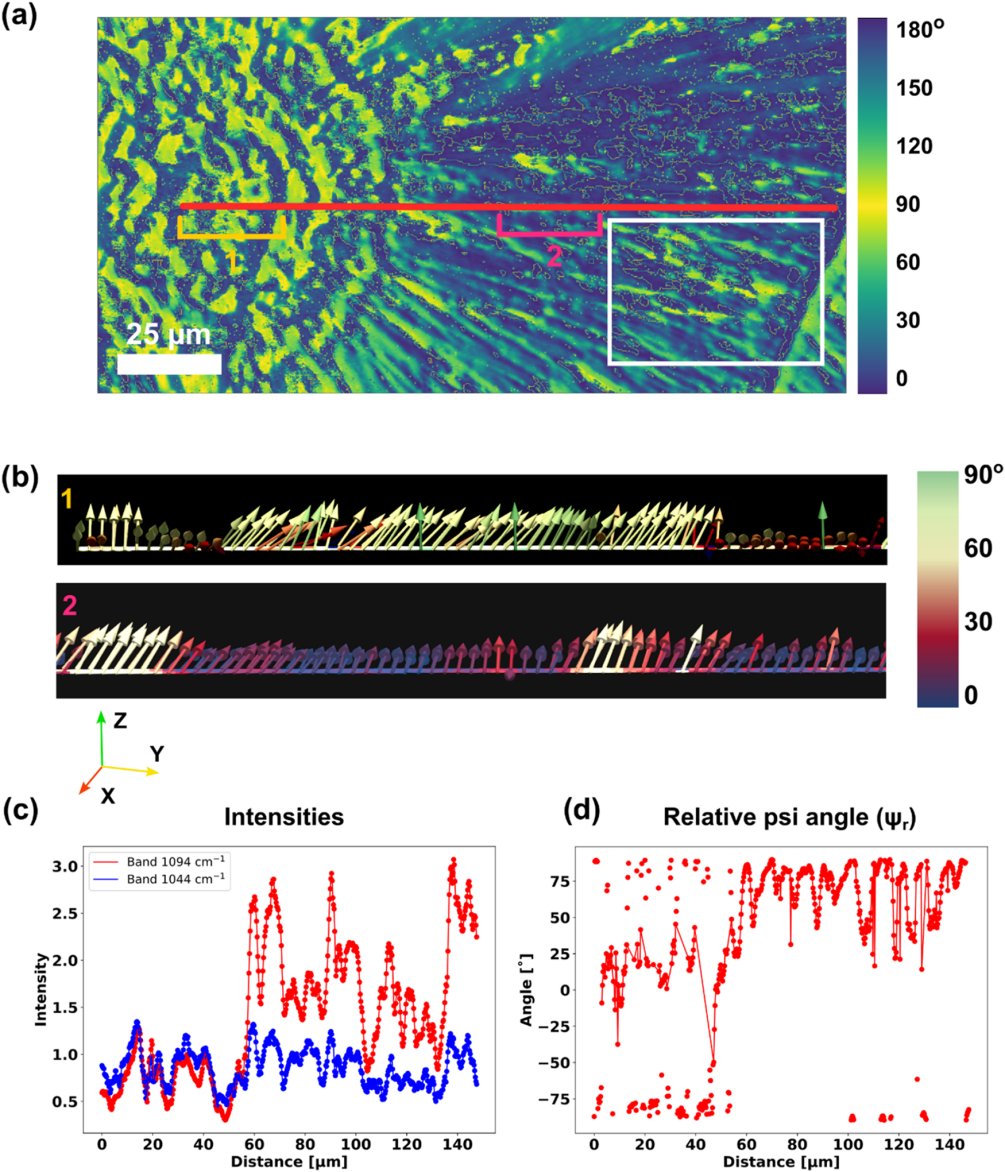
Illustrations of chain orientations of PLLA/PEG annealed in nonisothermal conditions. Azimuthal angle (ψ) map of the region marked with a rectangle in Figure 2c (a). 3D orientation of chains along the red line, limited to regions 1 and 2. The color of the rod indicates tilt (b). Intensity plots of 1094 and 1044 cm^–1^ bands for polarization 0° (horizontal, c) and ψ angle for lines marked on psi maps (d).

As in the previous case, based on calculations, we can hypothesize cyclic spherulite growth as the cause of the formation of a nest in the initial phase of spherulite growth. The crystal grows until the distance for diffusion of the necessary material does not exceed some critical value. After the consumption of available material, the height of the crystal starts to drop, and a valley is created. The material diffusion from the amorphous phase to the crystal front starts again due to the shorter distance, and the new peak occurs; the cycle repeats itself. Figure 5c shows the 1094 and 1044 cm^–1^ bands’ intensities along the red line from Figure 5a. Two phases of spherulite growth are easy to see. The two bands are perpendicular to each other, but for the ring-banded region, the polarization dependence was not observed. It suggests that the organization of the molecule is rather discrete and not perpendicular to the radius from the nucleation center. The intensity jumps match the rings. For the second region, the ψ map looked more similar to the previous spherulite. The angle changed continuously, in a radial manner. The in-plane orientation angle (ψ_r_) of the chains relative to the red line oscillated around 95°, which confirmed a perpendicular assembly of molecules to the axis of lamellae growth (Figure 5d). The out-of-plane orientation of chains was presented with the rods (Figure 5b, part 2). Mostly, the chains are directed perpendicular to the direction of growth and almost flat on the substrate; any kind of cyclic oscillation was not observed. However, the ψ map showed an irregular pattern, similar to the relative psi angle (ψ_r_) and intensities’ plots (Figure 5c,d). The calculation of the three orientation angles ruled out the lamella twisting as the cause of the oscillating values.

Type 2 exhibits a morphology different from the classic spherulite; the lamellae are not flat-stacked plates. The azimuthal angle (ψ) map projection reveals thin, branched lamellae filling the empty space. The details of lamellae’s assembling in three-dimensional space and the consideration of the branching mechanism are included in the next section.

The last film was prepared by isothermal annealing at 86 °C. In the DC image (Figure 2d), we can see many small, neighboring spherulites (Type 3). Crystallization at a temperature of 86 °C, close to the glass transition temperature, leads to an increase in the nucleation density. Figure 6a shows the azimuthal angle (ψ) map for the selected fragment. The morphology of the spherulite, the nucleation center, split fibrils resembling a plume, and the boundary between spherulites are distinguishable. The transition between one spherulite and the other spherulite is smoother than in the previous film, where the boundary was clearer. The azimuthal angle (ψ) changes radially, but the pattern shows a large irregularity. The 3D orientation representation in the form of rods was prepared for the pink section marked with number 1. A trend in the arrangement of the chains was observed; they were mostly almost perpendicular to the lamella growth axis, but the values of ψ and θ angles were oscillating irregularly (Figure 6b). Interestingly, according to the graphs, the intensities of the 1094 and 1044 cm^–1^ bands decreased with the growth of the spherulite (Figure 6c). This may indicate the depletion of material for building crystals, with the growth of the spherulite. Chain packing also became more chaotic with spherulite growth, as indicated by larger and larger deviations from the average value of the ψ_r_ angle (Figure 6d). It should also be emphasized that for the sample heated at 86 °C, the angle was lower than in the two previous films, and for many points did not exceed 75°, which may also be an indicator of a different mechanism of lamella assembling. The Type 3 spherulite contains dendrites that are oriented anisotropically.

**Figure 6:**
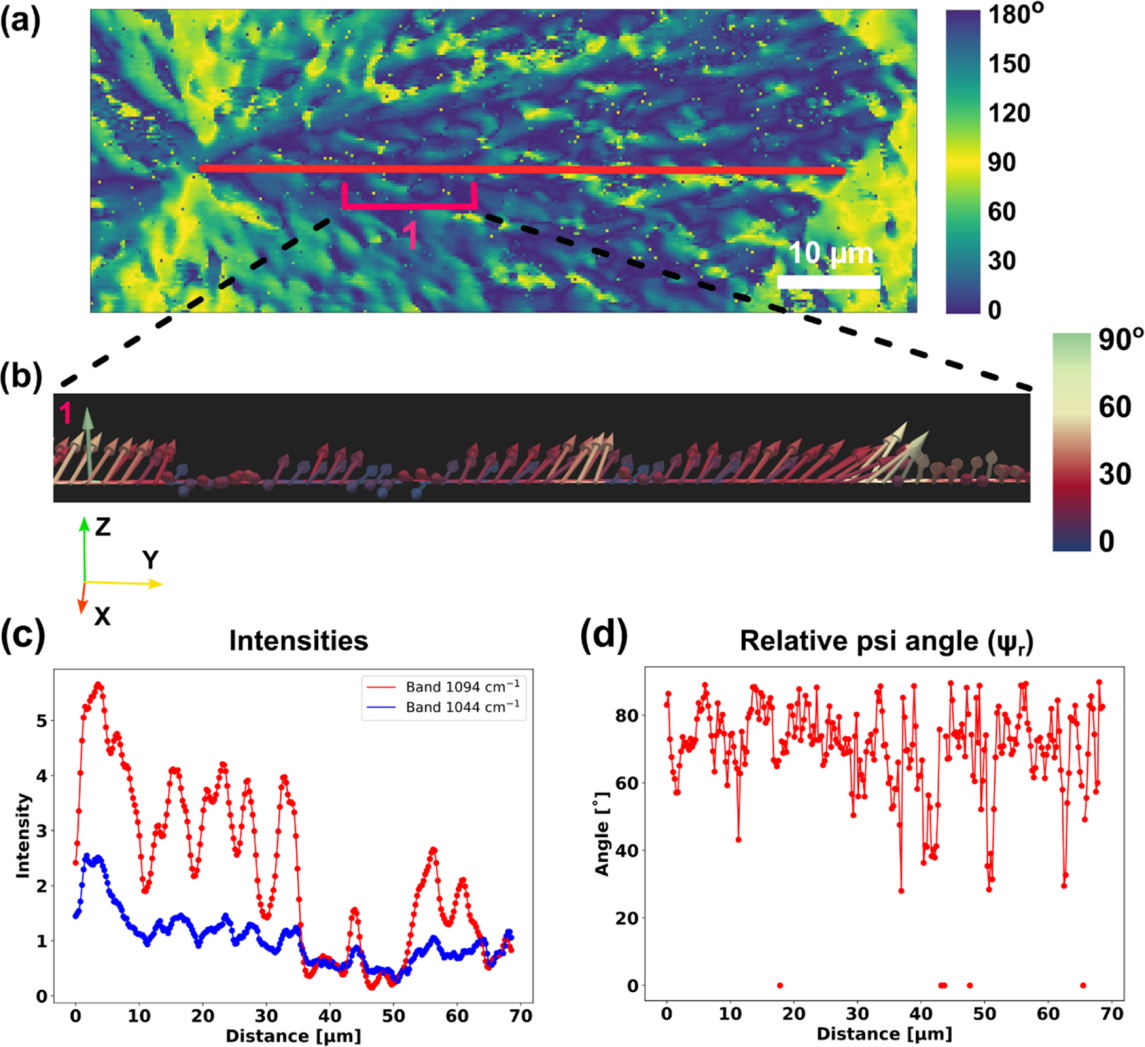
Illustrations of chain orientations of PLLA/PEG annealed at the first crystallization temperature. Azimuthal angle (ψ) map of the region marked with a rectangle in Figure 2d (a). 3D orientation of chains along the red line, limited to region 1. The color of the rod indicates tilt (b). Intensity plots of 1094 and 1044 cm^–1^ bands for polarization 0° (horizontal, c) and ψ angle for lines marked on psi maps (d).

### Variation of Spherulite Patterns under Different Conditions

To better visualize the morphology of spherulites with branched lamellae (Types 2 and 3), three-dimensional molecular orientation maps were prepared for selected fragments (Figure 7). In both cases, an unusual organization was observed, with crystallization temperature being the differentiating parameter. Type 2 spherulite exhibits a simple and compact pattern of branching lamellae. In the case of PLA/PEG crystallized at low temperatures, the bundle branches in a tree-like pattern, forming a spherulite. The lamellae are tightly packed. With the same composition and similar thickness, the morphology depends on the crystallization temperature. Based on the results presented in the previous section, we can formulate a preliminary hypothesis regarding the possible growth patterns of branched spherulites of PLLA with added PEG.

**Figure 7:**
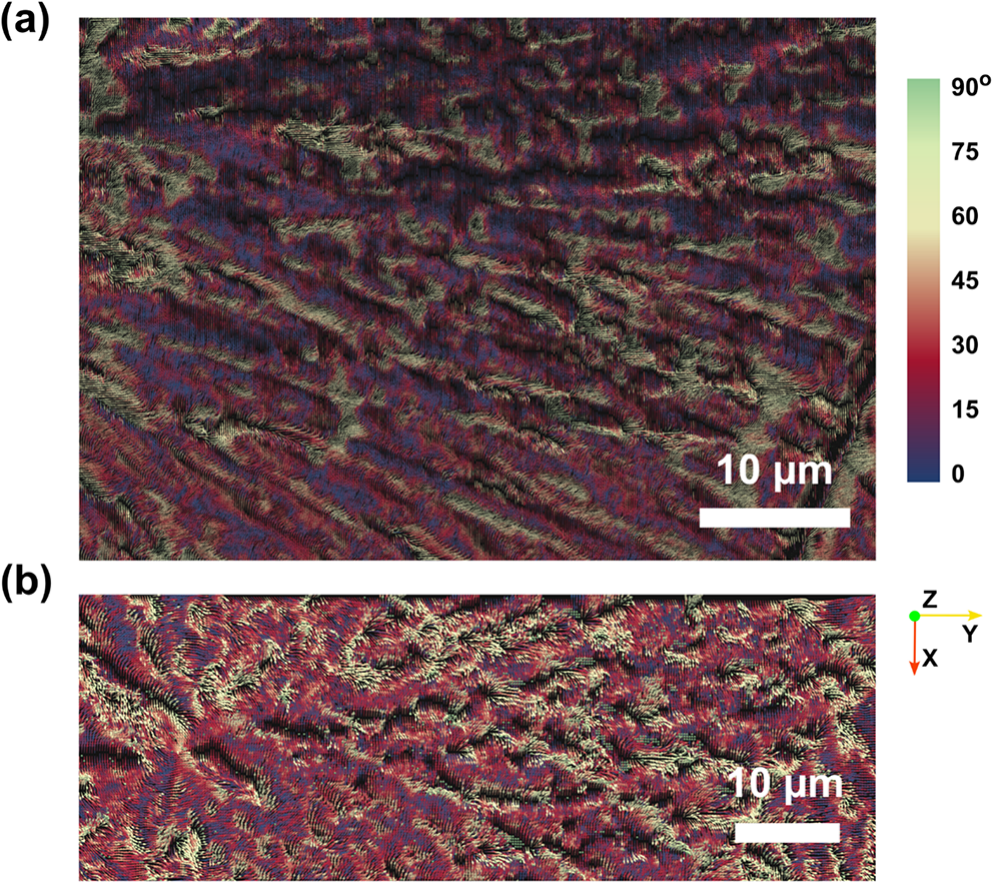
3D orientation vector map of branched spherulites: PLLA/PEG annealed in nonisothermal conditions (region marked with white frame in Figure 5a) (a). PLLA/PEG after crystallization at 86 °C (b). Top view.

The first 3D map revealed the nature of the Type 2 spherulite (Figure 7a) crystallized in the nonisothermal conditions. The illustration shows the top view of a small area marked with a white rectangle on the ψ map (Figure 5a) for better clarity. The morphology consisted of radially arranged fibrils with branching.

A shorter time for chain folding compared to high temperature crystallization implies more defective, thinner lamellae; however, macromolecules appeared to be well packed, creating a compact structure within a single entity. The addition of plasticizer and thermal conditions should be considered the source of this unusual morphology. The lamellar assembly suggests a high dynamic of the crystallization process taking place in nonisothermal conditions. Changes in crystallization rate and increased supercooling can lead to secondary crystallization^38^ and, subsequently, to the side branching, which was the source of the IR intensity and ψ_r_ oscillations. The temperature oscillations also promote this phenomenon. If the crystallization rate is high, macromolecules do not have enough time for self-alignment into linear lamellae, which favors branching.

Figure 7b shows the 3D orientation map of spherulite Type 3 crystallized at low temperature. The morphology consisted of multiply laterally branched, layered lamellae, which filled all possible space. The map indicates that it is a dendritic spherulite composed of tree-like structures that grow from a common nucleation core.

The second crystallization leads to the side branching of already existing lamellae.^38^ In this case, the high supercooling translates into a high thermodynamic driving force for crystallization, while chain mobility is limited due to the low crystallization temperature. However, this was enhanced by incorporating PEG into PLLA, as confirmed by DSC measurements (Figure 1a). The formation of dendritic side branches may stem from an imbalance between these factors. The diffusion of polymer chains toward the growth front is slower than the front’s advancement. This imbalance leads to growth front instability, where the boundary cannot remain smooth—it fragments into branched, tree-like structures (dendrites). Type 2 spherulites were branched but not dendritic. Crystallization conditions were nonisothermal. The effective growth front moves through regions where dendritic instability is transient. Growth is nonuniform, leading to branched spherulites. The greater chain diffusivity at higher temperatures helps stabilize the growth front; the instabilities are transient.

Confirmation of the presented hypotheses requires an in-depth study of the lamella organization and branching processes. Such results can be obtained by combining polarization analysis algorithms with real-time measurements or by cycling samples quenched at different crystallization times.

### Uncertainty of the O-PTIR Four-Polarization Method

Not all points provided correct ψ and θ values due to scattering at discontinuities and edges. Unlike in classical FT-IR microscopy, IR scattering in O-PTIR has a smaller impact on signal quality, though polarization-dependent scattering may still cause anisotropic artifacts. Scattering can reduce signal intensity and, if polarization-dependent, lead to anisotropic artifacts. Another significant source of artifacts in thin films is laser probe interference, which causes signal intensity oscillations related to sample thickness and optical path changes. The effect can mask the actual chemical contrast and lead to a nonlinear dependence of the signal on material thickness. Furthermore, different orientations can overlap in areas of discontinuity, violating the assumptions of the computational algorithm. Xu et al. discussed in their paper the influence of different orientations near discontinuities.^13^ The algorithm for calculating orientations is based on the assumption that the ODF function is cylindrical or uniaxial with respect to the local mean direction. Deviations can occur where different orientations overlap.

Artifacts remain a significant problem in IR thin film microscopy, which is why correction methods such as interference fringe modeling, background correction, and optimization of measurement conditions are used. In O-PTIR, the background profile is measured each time for different polarizations, and focusing is determined based on the maximum signal to limit the impact of changes in sample thickness.

## Conclusions

In this work, the complex layer systems in PLLA spherulites were explored. The O-PTIR experimental setup was modified with an automatic setup for precise polarization measurements. The three-dimensional orientation was calculated for each pixel collected every few hundred nanometers, and the linear 3D presentation from the nucleation center to the boundary allowed for tracing different paths of spherulite formation dependent on the crystallization conditions. The studies proved that the method is very sensitive to small structural changes and resolves the hierarchical structure of materials. We made the self-organization of chains dependent on the crystallization temperature and, thanks to the high spatial resolution of O-PTIR, we detected the second crystallization and branching in lamellae in spherulites that crystallized under dynamic conditions. Unlike FT-IR microscopy, photothermal O-PTIR microscopy is independent of the diffraction limit of IR light, and the samples do not require any special preparation, including substrates dedicated to IR measurements. The 3D orientation technique can have a big impact in other fields thanks to its compactness, providing information about chemical and structural properties at the same time. We see its future primarily in biological studies, such as tissues.
